# The importance of investigating frailty in chronic kidney disease

**DOI:** 10.1590/2175-8239-JBN-2023-E017en

**Published:** 2023-11-03

**Authors:** Adriano Luiz Ammirati

**Affiliations:** 1 Hospital do Rim, Unidade de Diálise, São Paulo, SP, Brazil.

The study by Bansal et al.^1^ published in the BJN was a cross-sectional study involving 90 adult outpatients from a hospital in New Delhi, who were divided into 3 groups according to stage of chronic kidney disease (CKD). The main objective was to examine the association between CKD and frailty. In addition, the relationship between glomerular filtration rate (GFR), urine albumin creatinine ratio (UACR), and comorbidities of the studied patients was evaluated.

The classic criteria for defining CKD were used. The studied variables were: frailty (Fried phenotype^2^), health deficits (pre-defined list of comorbidities and deficits based on a method described by Searle et al.^3^); depression (4-point depression scale); risk of falls (Get-up-and-go test); memory assessment (Folstein Mini Mental State Examination (MMSE), ability to perform activities of daily living (Barthel tests), and quality of life (World Health Organization Quality of Life Brief - WHOQOL-BREF). Blood and urine samples were collected to assess kidney disease.

The difference in the proportion of frail individuals was compared between different groups (GROUP A: stages 1 and 2 CKD; GROUP B: stage 3a CKD, and GROUP C: stages 3b and 4 CKD). The association between the number of health deficits and eGFR and UACR was also evaluated. Multiple logistic regression models were developed to explore the relationship between frailty and kidney disease and to identify factors that may predict frailty after adjusting for CKD.

The population that participated in the study consisted of young patients (49 ± 12.4 years old), predominantly female (57%) and equally divided into CKD groups (1/3 stages 1 and 2; 1/3 stage 3a, and 1/3 stages 3b and 4). The prevalence of frailty was 21.1%.

Comparing groups according to CKD stage, those in stages 3b and 4 had a higher proportion of frail people, and after adjusting for age, gender, depression, and cognitive impairment, patients in this group were nine times more likely to be frail than patients with better kidney function. Another marker of kidney damage, UACR, was significantly higher in frail patients than in non-frail ones.

Another important data show that patients with depression were six times more likely to be frail after adjustments. Furthermore, the number of deficiencies was higher in frail patients, so that having more than six deficits predicted frail patients (sensitivity of 0.79 and a specificity 0.72).

The prevalence of frailty in the present study (21%) was low compared to data available in the literature (37.5 to 42.6%)^4,5^, probably justified by the inclusion of relatively young patients, patients with early stages of CKD and outpatients, that is, functionally active people. It is important to highlight that the Fried phenotype is a validated method of frailty assessment that classifies patients as frail, pre-frail, or non-frail categories^2^, but it is less useful for grading the severity of frailty in populations where the prevalence of frailty is high^6^.

The study by Bansal et al.^1^ has limitations such as a cross-sectional design, a small number of patients, and single center with a population with little ethnic variation. However, the importance of the study was once again to show the association between renal dysfunction and frailty and to characterize frail patients who have more depression, more deficits, and consequently higher mortality and worse quality of life.

There is a clear consensus that the prevalence of CKD is increasing worldwide and that this pathology is associated with a series of comorbidities, symptoms, and limitations that impact people’s lives, especially in the advanced stages of renal dysfunction^7^. The frequency and burden of symptoms experienced by individuals undergoing dialysis like fatigue, pain, poor mood, dry skin, poor sleep, and muscle cramps are increasingly recognized^8^. Furthermore, the number of elderly dialysis patients is increasing worldwide, especially in developed countries^9^. In this context, it is understandable that the frailty phenotype, characterized by three or more of the five criteria of weakness, slowness, low level of physical activity, self-reported exhaustion, and unintentional weight loss, is frequent in the population with CKD, especially in advanced stages.

Better access to health care and improved dialysis procedures can lead to better survival of these patients. However, we must also address other aspects that are becoming increasingly evident, such as the frailty, depression, and low quality of life of this population^8^. In this context, the present study contributed to raise awareness of these important situations in patients with kidney dysfunction. Furthermore, there is a need to better understand why frailty occurs even in the early stages of CKD and why it is associated with adverse health outcomes in patients with established renal dysfunction.

## References

[1] Bansal L, Goel A, Agarwal A, Sharma R, Kar R, Raizada A (2023). Frailty and chronic kidney disease: associations and implications. J Bras Nefrol.

[2] Fried LP, Tangen CM, Walston J, Newman AB, Hirsch C, Gottdiener J (2001). Frailty in older adults: evidence for a phenotype. J Gerontol A Biol Sci Med Sci.

[3] Searle SD, Mitnitski A, Gahbauer EA, Gill TM, Rockwood K (2008). A standard procedure for creating a frailty index. BMC Geriatr.

[4] Mansur HN, Colugnati FA, Grincenkov FRDS, Bastos MG (2014). Frailty and quality of life: a cross-sectional study of Brazilian patients with pre-dialysis chronic kidney disease. Health Qual Life Outcomes.

[5] Lee SJ, Son H, Shin SK (2015). Influence of frailty on health-related quality of life in pre-dialysis patients with chronic kidney disease in Korea: a cross-sectional study. Health Qual Life Outcomes.

[6] Chowdhury R, Peel NM, Krosch M, Hubbard RE (2017). Frailty and chronic kidney disease: a systematic review. Arch Gerontol Geriatr.

[7] Kovesdy CP (2022). Epidemiology of chronic kidney disease: an update 2022. Kidney Int Suppl.

[8] Mehrotra R, Davison SN, Farrington K, Flythe JE, Foo M, Madero M (2023). Conference Participants. Managing the symptom burden associated with maintenance dialysis: conclusions from a Kidney Disease: improving Global Outcomes (KDIGO) Controversies Conference. Kidney Int.

[9] Saran R, Li Y, Robinson B, Abbott KC, Agodoa LY, Ayanian J (2016). US Renal Data System 2015 Annual Data Report: epidemiology of kidney disease in the United States. Am J Kidney Dis.

